# Survival of the Enveloped Virus Phi6 in Droplets as a Function of Relative Humidity, Absolute Humidity, and Temperature

**DOI:** 10.1128/AEM.00551-18

**Published:** 2018-05-31

**Authors:** Aaron J. Prussin, David Otto Schwake, Kaisen Lin, Daniel L. Gallagher, Lauren Buttling, Linsey C. Marr

**Affiliations:** a Via Department of Civil and Environmental Engineering, Virginia Polytechnic Institute and State University, Blacksburg, Virginia, USA; Rutgers, The State University of New Jersey

**Keywords:** influenza, coronavirus, SARS, MERS, humidity

## Abstract

Infectious diseases caused by enveloped viruses, such as influenza, severe acute respiratory syndrome (SARS), and Middle East respiratory syndrome (MERS), cause thousands of deaths and billions of dollars of economic losses per year. Studies have found a relationship among temperature, humidity, and influenza virus incidence, transmission, or survival; however, there are contradictory claims about whether absolute humidity (AH) or relative humidity (RH) is most important in mediating virus infectivity. Using the enveloped bacteriophage Phi6, which has been suggested as a surrogate for influenza viruses and coronaviruses, we designed a study to discern whether AH, RH, or temperature is a better predictor of virus survival in droplets. Our results show that Phi6 survived best at high (>85%) and low (<60%) RHs, with a significant decrease in infectivity at mid-range RHs (∼60 to 85%). At an AH of less than 22 g · m^−3^, the loss in infectivity was less than 2 orders of magnitude; however, when the AH was greater than 22 g · m^−3^, the loss in infectivity was typically greater than 6 orders of magnitude. At a fixed RH of 75%, infectivity was very sensitive to temperature, decreasing two orders of magnitude between 19°C and 25°C. We used random forest modeling to identify the best environmental predictors for modulating virus infectivity. The model explained 83% of variation in Phi6 infectivity and suggested that RH is the most important factor in controlling virus infectivity in droplets. This research provides novel information about the complex interplay between temperature, humidity, and the survival of viruses in droplets.

**IMPORTANCE** Enveloped viruses are responsible for a number of infectious diseases resulting in thousands of deaths and billions of dollars of economic losses per year in the United States. There has been a lively debate in the literature over whether absolute humidity (AH) or relative humidity (RH) modulates virus infectivity. We designed a controlled study and used advanced statistical modeling techniques specifically to address this question. By providing an improved understanding of the relationship between environmental conditions and virus infectivity, our work will ultimately lead to improved strategies for predicting and controlling disease transmission.

## INTRODUCTION

Infectious diseases caused by enveloped viruses, such as influenza viruses and the coronaviruses responsible for severe acute respiratory syndrome (SARS) and Middle East respiratory syndrome (MERS), cause thousands of deaths and billions of dollars of economic losses per year (1–3). Both influenza and SARS exhibit seasonality, with increased incidence during wintertime in temperate regions and perhaps during the rainy season in tropical regions (4–10). Understanding infectious disease transmission is a complex problem due to the many factors involved, such as environmental conditions, human activity (e.g., more crowding in buildings during wintertime), hygiene, and host susceptibility. Environmental conditions are particularly compelling to examine when considering seasonality due to clear changes in weather throughout the year in temperate regions.

Although the root cause of influenza and SARS seasonal patterns remains elusive, a confluence of recent epidemiological, animal, and laboratory studies has found a relationship between environmental conditions—temperature, absolute humidity (AH), relative humidity (RH), and/or rainfall—and influenza virus incidence, transmission, or survival (10–18). The first studies of the effect of RH on influenza virus survival emerged in the 1940s (19, 20), and more followed (21–24). The results indicated either a monotonically decreasing relationship between virus survival and humidity (i.e., lower survival at high humidity) or a “U-shaped” relationship with reduced survival at mid-range humidities. Two studies described similar relationships for the SARS coronavirus and its surrogates (25, 26). The seemingly conflicting results may be due to the use of different media for virus suspensions (13).

There has been considerable debate over whether AH or RH modulates influenza virus survival and transmission. AH describes the mass of water vapor per volume of air (i.e., the concentration of water vapor in air), while RH describes the ratio of the actual concentration of water vapor to the maximum possible concentration, which varies strongly with temperature. Shaman and Kohn (12) have concluded that the relationship is stronger with AH than with RH. According to their analysis of virus survival in aerosols (24) and transmission in guinea pigs (16), AH explains 50% and 90% of the variability in influenza virus transmission and survival, respectively, whereas RH explains only 12% and 36%, respectively, of the variability. In a study examining influenza virus survival in droplets at elevated temperatures (55 to 65°C), McDevitt et al. (27) also reported that AH is a better predictor than RH for virus inactivation. Shaman et al. (15) have modeled influenza-related mortality and suggested that changes in AH alone are the root cause of seasonal trends, whereas RH is a poor predictor. A causality analysis of global flu incidence data has shown that AH is a stronger driver than RH (18).

Despite the agreement among these studies that AH is more important than RH for influenza virus survival and transmission, we contend that the debate is not settled. The relationship between survival and humidity is confounded by the fact that higher values of AH can only be achieved at higher temperatures, which are known to accelerate virus inactivation (28). Epidemiological studies involving humidity have necessarily relied on data from outdoor weather stations, but almost all transmission probably takes place indoors, where people spend 90% of their time (29). Indoor RH is very strongly correlated with outdoor AH during the winter heating season (30), so we cannot yet rule out RH as a predictor for influenza incidence. Finally, we have proposed a mechanistic explanation for the relationship between virus survival with RH that revolves around the extent of evaporation and the resulting increase in solute concentrations in droplets (13), but we are not able to identify one for AH.

Although many factors affect disease transmission, we designed a study explicitly to discern whether AH or RH is a better predictor of virus survival in droplets, while also considering temperature in order to advance a mechanistic understanding of the relationship between virus inactivation and environmental conditions. We exposed an enveloped virus to seven environmentally relevant RHs and four temperatures to test the hypothesis that RH is more important than AH in modulating virus infectivity. Additionally, we hypothesize that over the range of temperatures typically found indoors (19 to 25°C), temperature does not have a significant effect on infectivity. The resulting data set is larger than has been obtained previously and enables a robust analysis that goes beyond simple linear regression. Due to the challenges and biosafety concerns of working with the influenza virus and coronavirus, this study employed the enveloped bacteriophage Phi6, which has been suggested as a surrogate for the influenza virus (31, 32) and SARS coronavirus (33). Our results provide novel information about the complex interplay between temperature, humidity, and the survival of viruses in droplets. This methodology may serve as a model for future efforts to study viruses that require more stringent biosafety protocols and to assess whether the results shown here are generalizable to other viruses.

## RESULTS

### Phi6 infectivity versus RH.

**FIG 1:**
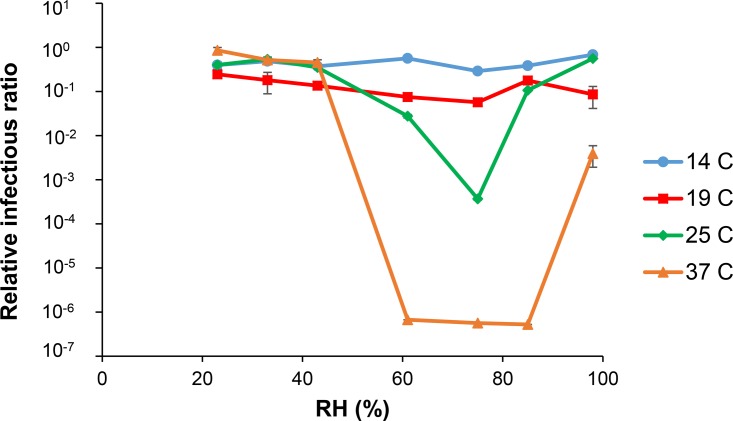
Relationship between RH and Phi6 infectivity at 14°C, 19°C, 25°C, and 37°C. AH ranged between 2.7 and 41.6 g · m^−3^. Standard error bars (*n* = 3) are shown; they are small enough to not be visible for many points.

We measured the infectivity of Phi6 in droplets exposed to RHs of 23%, 33%, 43%, 61%, 75%, 85%, and 98% at temperatures of 14°C, 19°C, 25°C, and 37°C. As shown in Fig. 1, virus infectivity, defined as the ratio of the concentration of PFU derived from the exposed droplets to the concentration of PFU in a control, depended on both RH and temperature. A one-way analysis of variance (ANOVA) of all data points showed there was a significant (*P* = 0.0002) influence of temperature on Phi6 infectivity; however, at 14°C and 19°C, virus infectivity remained high across all RHs tested. There was a significant effect of RH (*P* < 0.0001) on Phi6 infectivity at each temperature tested, except at 19°C (*P* = 0.0528). At 25°C and 37°C, we observed the same U-shaped RH dependence that Yang et al. (13) reported for influenza A virus in medium containing very little protein or none at all. At 25°C and 37°C, Phi6 infectivity was highest at RHs of less than ∼60% and greater than 85%. At 37°C and between ∼60% and 85% RH, there was an ∼6-log reduction in infectivity. At 25°C, the relative infectious ratio was 3 log lower at 75% RH than at 98% RH. For comparison, at 37°C, there were ∼6-log and ∼2-log losses in Phi6 infectivity at 75% and 98% RH, respectively, a difference in infectivity of 4 log even though there was only an ∼20-point difference in RH. To put these numbers into broader perspective, most environmental health and safety programs consider biological disinfection to occur when there is a 4- to 5-log reduction in starting microbial concentrations.

### Phi6 infectivity versus AH.

**FIG 2:**
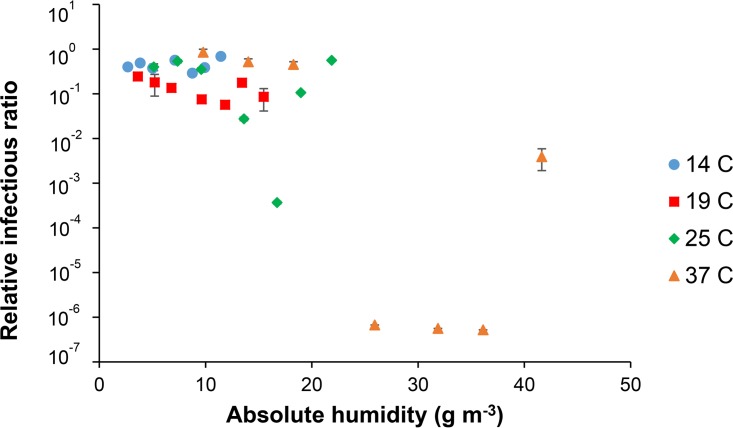
Relationship between AH and Phi6 infectivity at 14°C, 19°C, 25°C, and 37°C. RH ranged between 23% and 98%. Standard error bars (*n* = 3) are shown; they are small enough to not be visible for many points.

We tested the infectivity of Phi6 in droplets exposed to AHs ranging between 2.7 and 41.6 g · m^−3^ with temperatures ranging between 14°C and 37°C. As shown in Fig. 2, at an AH of less than approximately 22 g · m^−3^, the loss in infectivity was generally less than 2 log; however, when the AH was above approximately 22 g · m^−3^, the loss in infectivity was typically greater than six orders of magnitude. All of the AHs above 22 g · m^−3^ correspond to a temperature of 37°C. Using a one-way ANOVA, we found that there was a significant effect of AH on infectivity (*P* < 0.0001).

### Phi6 infectivity versus temperature.

**FIG 3:**
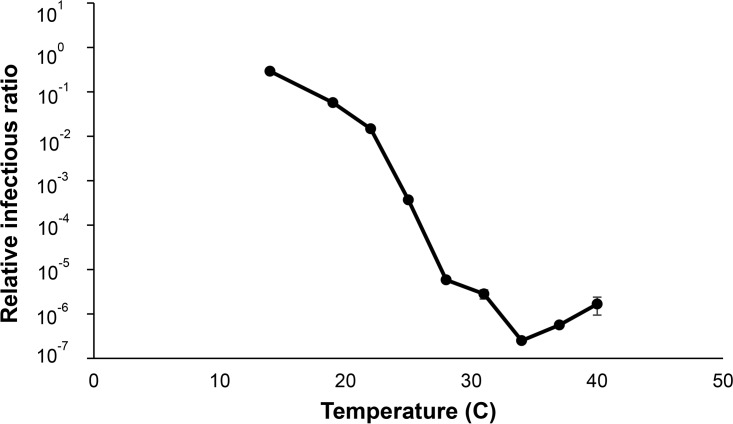
Relationship between temperature and Phi6 infectivity at 75% RH. AH ranged between 8.8 and 37.1 g · m^−3^. Standard error bars (*n* = 3) are shown; they are small enough to not be visible for many points.

To examine the effect of temperature on Phi6 infectivity (Fig. 3), we held the RH constant at 75% and tested virus infectivity at nine temperatures: 14°C, 19°C, 22°C, 25°C, 28°C, 31°C, 34°C, 37°C, and 40°C. We observed the highest infectivity (28.97% ± 2.45%) of Phi6 at the lowest temperature tested (14°C), and there was a reduction in infectivity of more than 6 log when the temperature was increased to 34°C. Between 14°C and 34°C, the virus infectivity decreased exponentially as the temperature increased. A one-way ANOVA revealed that temperature had a significant effect on Phi6 infectivity across all temperatures below 34°C (*P* < 0.0001); however, at temperatures above 34°C, there was no effect of temperature on virus infectivity (*P* = 0.1293). For perspective, medical equipment meets the sterility assurance level when there is a 6-log reduction in the number of microorganisms.

### Environmental variables modulating Phi6 infectivity.

Multiple linear regressions of infectivity against temperature, RH, and AH revealed that the slopes for temperature and AH were statistically significant, but that for RH was not. Partial *F* statistics confirmed that the addition of RH to a model already containing AH did not improve the model. However, the overall fit of the multiple regression model was poor, with an *R*^2^ of 0.34. Using a multiple regression with the dependent variable of the log of infectivity improved the *R*^2^ to 0.64. For this model, both AH and RH were statistically significant, based on partial *F* statistics, but temperature was not.

**FIG 4:**
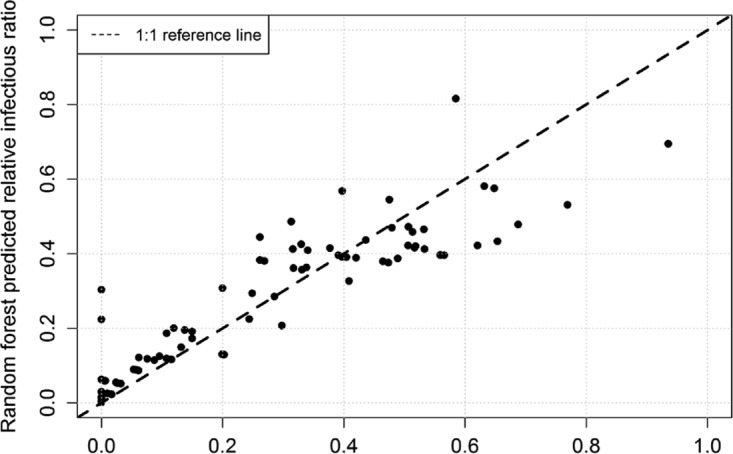
Predicted versus observed infectivity using random forest model.

The random forest was the most successful model applied. Figure 4 depicts the predicted infectivity versus the observed infectivity and shows that the percentage of explained variance (pseudo *R*^2^) was 0.83. The relative importance of the variables, measured as the increase in the mean squared error when the variable was deleted from the model, was RH > temperature > AH. The normalized variable importance was 1.00, 0.99, and 0.84 for RH, T, and AH, respectively. The random forest model applied to a log_10_ reduction in infectivity (instead of just relative infectivity) did not perform as well; the percentage of explained variance was 0.59.

### Infectivity in droplets versus aerosols.

**FIG 5:**
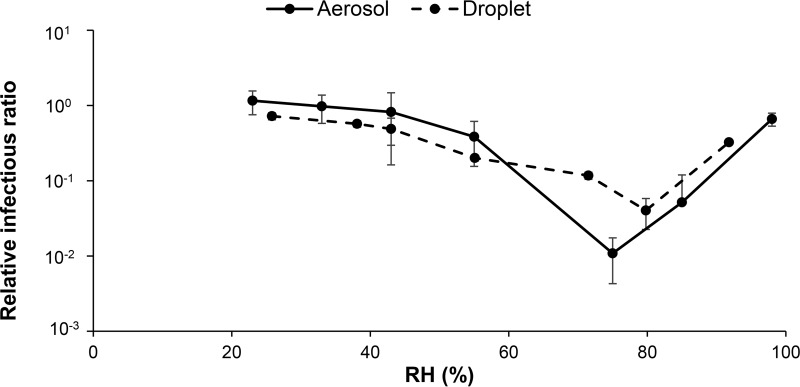
Relationship between RH and Phi6 infectivity in aerosols and droplets aged for 1 h at 22°C. The correlation coefficient is 0.98, and there is no significant difference between the two distributions (*P* = 0.095).

As some viral diseases are transmitted via the airborne route, the inevitable questions are whether virus inactivation is similar in stationary droplets and suspended aerosols and whether droplets, which are much easier to work with, can be used as a surrogate for aerosols in the study of virus inactivation. We measured virus stability in both droplets and aerosols under the same conditions, and the relationship between infectivity and RH was essentially the same in both (Fig. 5). The correlation coefficient was 0.98, and a Kolmogorov-Smirnov test indicated that there is no significant difference between the two distributions (*P* = 0.095).

## DISCUSSION

Many factors affect disease transmission, and we designed a study examining one piece of the puzzle to advance a mechanistic understanding of the relationship between virus inactivation and environmental conditions. The results of this study apply to transmission by droplets and aerosols that remain exposed to the environment on the time scale of many minutes to hours, or long enough for environmentally mediated inactivation to occur. This study was designed specifically to compare the importance of RH, AH, and temperature as predictors of enveloped virus survival in droplets. Ultimately, we hope this work can help explain the trends in diseases caused by enveloped viruses and lead to improved disease control strategies. Our results suggest that in determining virus infectivity, the interaction among RH, AH, and temperature is complex and not easily captured by linear or log-linear regressions. Shaman and Kohn (12) found that AH was more important than RH in determining virus survivability, but they only evaluated each variable singly. The comparison is confounded by temperature, because higher values of AH are only achievable at higher temperatures, and virus infectivity is reduced at higher temperatures regardless of humidity. Additionally, their data set only contained 11 RH/AH/temperature combinations, compared to 28 combinations in our study.

When we performed a multiple regression analysis on our data set, we reached the same conclusion as Shaman and Kohn (12) (i.e., that AH was a better predictor of virus infectivity), but the overall fit was poor, with all three variables together only explaining 34% of the variability. The log-linear regression performed better but found that both RH and AH together were important in predicting infectivity. The random forest model fit the data best of all the approaches used (*R*^2^ = 0.83). The random forest model suggested that RH was the most important factor in controlling Phi6 infectivity in droplets. Random forests have the advantage that they do not need a prespecified model to evaluate as regressions do. Random forests develop the model based on the data and can thus provide improved results for models based on correlated interacting variables (34). They also directly supply a variable importance. These findings suggest that going forward, researchers need to take care in choosing the most appropriate model for statistical analyses when working with colinear data sets, as the model chosen can lead to different conclusions even when working with the same experimental data.

The effect of RH, AH, and temperature on the infectivity of viruses in droplets is complex and is likely due to the interaction between droplet physicochemical characteristics and virus physiology. We previously proposed a mechanistic explanation for the relationship between influenza survival in droplets and RH, with partial evaporation and subsequent concentration of solutes explaining some of the results observed (13). Briefly, when virus is released into the environment as part of a respiratory fluid droplet, RH, not AH, controls how much water evaporates from the droplet until it reaches equilibrium with the surrounding air, as described by the Kohler equation. The amount of water lost affects salt and protein concentrations, and potentially pH, in the droplet, and changes in these properties could affect virus survival, although more work is needed to understand these effects on a physiological level.

This study employed 1-μl droplets that are relevant to disease transmission by “large droplets,” but they are much larger than what are considered “aerosols.” While someone who is infected might cough or sneeze out droplets larger than the ones in this study, that person will also expel many other smaller droplets and aerosols (35, 36). Larger droplets can take a long time to evaporate, especially at high humidity, while very small droplets are able to evaporate in milliseconds (37). One potential explanation for why we observed a significant effect of temperature on virus infectivity at high humidities (>60%) and not lower humidities (Fig. 1) is because at lower humidities, the droplets evaporated and the virus was potentially in a “desiccated” state that was unaffected by changes in temperature. It is well known that other biological materials (e.g., bacterial spores, fungal spores, and nematodes) are able to survive in a desiccated state, and in fact, desiccation is used for the long-term preservation of microbial samples (38, 39). Greiff and Rightsel (40) showed that removing the residual moisture from influenza virus to ∼2% enabled the virus to maintain its titer level for 13 to 20 days at 28°C and 2.5 to 3.5 days at 45°C. However, “overdrying” and removing too much moisture produced a less protective effect; when dried to a residual moisture of 0.4%, the influenza titer was only maintained for 2 days at 28°C. In our study, at higher humidities (>60%), the droplets remained wet, and thus virus infectivity was potentially affected by both temperature-mediated exponential decay as defined by the Arrhenius equation and changes in droplet chemistry due to the partial evaporation of water. We speculate that at a very high humidity (>85%), virus decay is modulated by temperature changes (since the droplet is at near-physiological conditions), while at a mid-range RH (60 to 85%), virus decay is due to changes in droplet chemistry due to evaporation. A full mechanistic explanation may be revealed by future research examining the chemistry of evaporating droplets and the physiological effect on virus.

Considering that we spend more than 90% of our time indoors (29), there may be an opportunity to moderate disease transmission by controlling temperature and RH in the built environment. Contrary to our initial hypothesis that temperature over the range of that typically found indoors (19 to 25°C) does not have a significant effect on virus infectivity, we found that this small range can have profound effects on infectivity at a fixed RH of 75%. Over this range, virus infectivity decreases exponentially with increasing temperatures (Fig. 3). During the wintertime, when most diseases caused by enveloped viruses reach peak incidence, building temperatures are typically lower than during the summertime in order to save energy and money. However, lower indoor temperatures create a more favorable environment for virus survival. For example, if the RH is constant at 60% in a building, our results predict that virus infectivity would be ∼7.5% at 19°C but only ∼2.8% at 25°C, a >2-fold reduction in infectivity.

Similarly, by controlling RH in the built environment, we might be able to create conditions that are less favorable for virus survival in droplets. During wintertime in temperate regions, the RH of buildings tends to be very low. Reynolds et al. (41) measured the RH in six commercial buildings in the midwestern United States between November and April, and the RH was less than 25% in all buildings studied. On the basis of our results, a low RH is favorable for virus survival. Thus, by increasing both RH and temperature, we might be able to inhibit virus survival and thus reduce disease transmission via droplets in buildings such as homes, offices, schools, and hospitals.

Before controlling RH and temperature in their buildings, building managers should take caution and consider potential tradeoffs. Multiple factors, including occupant comfort, energy use and costs, and implications on other microorganisms, should be considered when selecting RH and temperature targets (42). For example, results from this study suggest that the optimal conditions to hinder enveloped virus survival would be 37°C and 85% RH; however, from a comfort (and energy/cost savings) standpoint, this temperature is obviously not reasonable. Additionally, this RH is likely to promote the growth and survival of some bacterial and fungal species (43, 44). Mold grows best when the RH is above 60%, and the U.S. Environmental Protection Agency (EPA) recommends keeping indoor RH between 30% and 50% (45). Figuring out the optimal indoor conditions to balance comfort, energy savings, and control of total microbial growth is a complex problem that will require an interdisciplinary effort between microbiologists, engineers, and building scientists going forward.

There are some potential limitations to this study. First, to overcome some of the challenges and technical limitations of working with human pathogens, previous research has suggested that bacteriophage Phi6 is an appropriate surrogate for infectious enveloped viruses (e.g., influenza, and SARS) (31–33). The advantages of working with Phi6 include its biosafety level 1 (BSL-1) biohazard rating, the ability to rapidly and accurately quantify virus infectivity through plaque assays, and its extensive characterization. Phi6, influenza, and coronaviruses are all lipid-enveloped RNA viruses that are ∼100 nm in diameter. One important molecular difference is that Phi6 contains double-stranded RNA, while influenza and coronaviruses contain single-stranded RNA. It is unlikely, but possible, that this molecular difference could lead to differences in infectivity as a function of environmental conditions; however, this requires future research. Researchers recently evaluated the suitability of Phi6 as a universal surrogate and cautioned that it might not be an appropriate surrogate for all enveloped viruses in all environments (46). Whereas only 1 to 10% of influenza virus particles are infectious (47, 48), nearly all bacteriophage particles are thought to be infectious, and so infectivity may be much more labile for influenza virus than for the surrogate. As this study focuses on the patterns in decay as a function of temperature and humidity rather than the absolute value of decay, differences in lability are hopefully minimized. Additionally, one might argue that droplets are not representative of aerosols, but we have observed similar results for Phi6 infectivity versus RH in both stationary droplets and suspended aerosols (Fig. 5). Finally, Phi6 was suspended in tryptic soy broth (TSB; growth medium for Phi6) for these experiments, which is very simple compared to the respiratory fluid that typically surrounds enveloped viruses that have been expelled into the environment. The medium composition can have a profound effect on virus infectivity (13). We have previously hypothesized that mucin glycoproteins might provide a protective effect for viruses against changes in RH (13). Understanding how different media protect viruses from changes in environmental conditions should be a future research priority.

This study examined only one aspect, the effect of humidity and temperature on virus stability in droplets and aerosols, of many that control the transmission of infectious disease. Other environmental factors, such as sunlight, wind, and rainfall, are also known or suspected to affect transmission. Seasonal variations in contact rates between infected and susceptible hosts, such as an increased time spent indoors during the winter, have been suggested to explain the seasonality of influenza (9). Host susceptibility plays a very important role in disease transmission, and immune function may vary seasonally with temperature, humidity, and solar radiation (9). Our results address one component of the disease transmission process and ultimately could be incorporated into a comprehensive model that considers all factors involved in transmission.

Our results provide insight into how RH, AH, and temperature affect enveloped virus droplet infectivity, and there are many directions for future work. First, as we have shown that the choice of statistical model has a significant effect on the conclusions, we recommend reanalyzing the data collected from previous studies of the effect of RH versus AH on enveloped virus infectivity using different statistical models beyond linear regression. Future efforts are also needed to study viruses that require more stringent biosafety protocols to assess whether the results shown here are generalizable to pathogenic viruses. Additionally, we recommend that future studies use more physiologically relevant media (e.g., respiratory fluid) to test the hypotheses that (i) medium composition has an effect on virus infectivity when exposed to different environmental conditions and (ii) glycoproteins provide a protective effect for virus in droplets against changes in RH and temperature. Finally, an interdisciplinary research effort is needed to define optimal indoor environmental conditions. Ultimately, these efforts will lead to improved strategies to control infectious disease transmission in the built environment.

## MATERIALS AND METHODS

### Virus and host.

We used the bacteriophage Phi6 (kindly provided by P. Turner of Yale University, New Haven, CT) as a surrogate for influenza virus and other enveloped viruses in these experiments (31). The host for Phi6 was Pseudomonas syringae pv. phaseolicola, and we cultivated it on tryptic soy agar (TSA). We prepared stock solutions of virus by suspending propagated Phi6 in TSB at concentrations of approximately 10^8^ to 10^10^ PFU · ml^−1^. We stored working stocks of Phi6 at 4°C and long-term stocks at −80°C.

### Chamber to control RH and AH.

To control humidity, we used a chamber as previously described (13). We placed two polyethylene petri dishes filled with 10 to 20 ml of a saturated salt solution into the bottom of a glass desiccator. Saturated solutions of potassium acetate, magnesium chloride, potassium carbonate, magnesium nitrate, sodium chloride, potassium chloride, and potassium sulfate provided stable RH at 23%, 33%, 43%, 61%, 75%, 85%, and 98%, respectively. We also placed a battery-powered computer fan into the chamber to promote airflow and a more rapid establishment of an RH equilibrium.

We regulated temperature by incubating the chamber in temperature-controlled rooms (14°C and 37°C), an incubator (25°C), or on a benchtop (19°C). To isolate the effect of temperature at a single RH of 75%, we incubated the chamber at additional temperatures of 22°C, 28°C, 31°C, 34°C, and 40°C. We selected 75% RH for this experiment for the following reasons. First, there were distinct differences in infectivity versus temperature at 75% RH (Fig. 1). Second, 75% was near the middle of the range (60 to 85% RH) in which we saw the largest virus inactivation. Third, we were able to test a full range of temperatures at 75% RH. A sensor (HOBO Temp/RH 2.5% data logger, Onset Computer Corporation, Bourne, MA) placed inside the chamber logged the temperature and RH. We considered an experiment valid if the temperature varied by no more than ±1°C and the RH by no more than ±2% during the incubation period. To determine AH, we calculated the saturation vapor pressure of water as a function of temperature (49), converted to units of grams per cubic meter, and multiplied by RH.

Before placing samples into the chamber, we allowed the chamber to reach equilibrium at the desired RH and temperature. When we briefly opened the chamber to place the samples inside, the temperature did not shift, but there was a small change in RH. However, the equilibrium was reestablished within approximately 5 to 10 min under all experimental conditions. Following every test, we washed the chamber with distilled water and ethanol to remove any salt residue.

### Experimental procedure.

For each of three biological replicates under each experimental condition, we spotted 10 1-μl droplets of Phi6 at a concentration of 10^8^ to 10^10^ PFU · ml^−1^ onto one well of a six-well, untreated, polystyrene cell culture dish (Greiner Bio-One CELLSTAR cell culture multiwell plate). We then placed the cell culture dish into the chamber, placed the lid on the chamber with a thin layer of vacuum grease to create a seal, and moved it to the appropriate location for incubation at the desired temperature. The entire process of diluting the working stock, spotting the droplets, and placing the cell culture dish into the RH chamber took place in a biological safety cabinet (BSC) at approximately 20°C.

Immediately following a 2-h incubation period, we returned the chamber to the BSC and removed the cell culture dish. We added 500 μl of TSB to each well containing droplets, which we resuspended via pipetting and then placed into separate cryovials for each well's solution. We placed the vials in a −20°C freezer for 20 min before moving them to a −80°C freezer for long-term storage. Prior to assaying these samples, we thawed them at room temperature before sampling and refreezing.

Samples for each experimental condition included negative-control cultures containing 500 μl of Phi6 stock solution. We stored these controls in polyethylene tubes and did not spot them as droplets but otherwise incubated them in the RH chamber and stored and assayed them identically to the test cultures.

### Determining Phi6 infectivity.

We performed plaque assays to quantify infectious Phi6 in the form of PFU (PFU · ml^−1^) for stock, control, and test cultures. For the plaque assays, we first performed serial dilutions of our working stock in TSB medium. We then mixed 50 μl of the diluted Phi6 with 250 μl of overnight cultured P. syringae, with the coculture inoculated into 6.5 ml of TSB soft agar prewarmed to 50°C. We quickly, but fully, hand-shook the mixture and poured it onto TSA plates, which we rotated by hand to allow the top agar to form a smooth even layer on the TSA. After drying, we sealed the plates in Parafilm and incubated them at 25°C for 24 h. To determine the concentration of infectious Phi6 in a sample, we multiplied the number of observed plaques by the appropriate dilution coefficient. We calculated the relative infectious ratio after aging Phi6 droplets at a given temperature, RH, and AH as relative infectious ratio (%) = *N_E_*/*N_C_*, where *N_E_* and *N_C_* are the measured Phi6 concentrations (PFU · ml^−1^) for the exposed and control samples, respectively.

### Droplets as a model for aerosols.

To examine whether the droplets serve as a valid model for aerosols, we examined virus decay in both under the same environmental conditions. We performed the droplet experiments as described above, with the exception of aging for 1 h to mimic the aerosol experiments. We studied the effect of RH on Phi6 in aerosols using a rotating drum (50). Briefly, a Collison nebulizer aerosolized Phi6 from a suspension in TSB into the drum at a controlled RH. We collected an unaged aerosol sample onto a gelatin filter and a sample that had been aged for 1 h onto another gelatin filter. To determine the infectivity of Phi6 in aerosols, we dissolved the filters in TSB and performed plaque assay on the samples. We corrected the results for losses in the rotating drum (50).

### Statistical analysis.

We determined the average Phi6 infectivity and the standard error from triplicate samples for each RH-temperature combination tested. We calculated all statistics in R version 3.4.2 (51) and JMP Pro 13. To calculate random forests, we used the randomForest package version 4.6-12 in R. The calculation was based on 50,000 trees and the default one variable used at each split, given the three explanatory variables available. We fitted virus infectivity as a function of temperature as exponential decay and calculated a coefficient of determination using Excel. We used a one-way ANOVA to test for significant differences (*P* < 0.05) in Phi6 infectivity between different temperatures, RH, and AH in JMP Pro 13.
